# The role of breastfeeding as a protective factor against the development of the immune-mediated diseases: A systematic review

**DOI:** 10.3389/fped.2023.1086999

**Published:** 2023-02-16

**Authors:** Amna A. Alotiby

**Affiliations:** Department of Hematology and Immunology, Faculty of Medicine Umm Al-Qura University, Makkah, Saudi Arabia

**Keywords:** allergy, breastfeeding, immunity, diabetes, infants, lactation

## Abstract

**Introduction:**

Breast milk is rich in nutrients and immunological factors capable of protecting infants against various immunological diseases and disorders. The current systematic review has been framed with the objective of studying the role of breastfeeding as a protective factor against the development of immune-mediated diseases.

**Methods:**

The database and website searches were performed using PubMed, PubMed Central, Nature, Springer, Nature, Web of Science, and Elsevier. The studies were scrutinized based on the nature of participants and the nature of disease considered. The search was restricted to infants with immune-mediated diseases such as diabetes mellitus, allergic conditions, diarrhoea, and rheumatoid arthritis.

**Results:**

We have included 28 studies, out of which seven deal with diabetes mellitus, two rheumatoid arthritis, five studies about Celiac Disease, twelve studies about allergic/ asthma/wheezing conditions and one study on each of the following diseases: neonatal lupus erythematosus and colitis.

**Discussion:**

Based on our analysis, breastfeeding in association with the considered diseases was found to be positive. Breastfeeding is involved as protective factor against various diseases. The role of breastfeeding in the prevention of diabetes mellitus has been found to be significantly higher than for other diseases.

## Introduction

During the first few years of a person's life, the immune system may be readily reshaped, which is important for achieving full protection against infections and the ability to tolerate non-harmful environmental substances to an adequate degree (1). Breastfeeding is geared to the needs of the newborn, and it may compensate for the relative inadequacy of the host defence by delivering substantial quantities of both nonspecific and pathogen-specific secretory IgA (sIgA) (2). Breastfeeding is adapted to the requirements of the infant (1). These antibodies, which are generated as a result of the earlier exposure to infectious agents by the mother, are capable of binding to potentially dangerous pathogens and rendering them inactive (1). Breast milk includes various additional nonspecific components that have antimicrobial properties or give protection to the newborn *via* different channels (3). These substances are present in addition to the antibodies that are present in breast milk (3). It's possible that the immunological, hormonal, enzymatic, trophic, and/or bioactive substances that are found in breast milk might provide some degree of passive protection (4). Other components, including as macrophages and leukocytes, which are predominantly present at the start of breastfeeding, may have a stronger modulatory influence on the immune system of the neonate and give further protection (5).

Breastfeeding has been regarded as the major protective factor in the lives of infants. The primary milk produced by the mothers is referred to as colostrum, which is found to be rich in immunologically active molecules and various nutrients and vitamins that are absolutely necessary for the growth of the infants (6). Infant's breastfed during their early life have developed immunity against various diseases considerably (7). The infants provided with breastfeeding have also been found to be devoid of malnutrition conditions (8). According to the World Health Organization, breastfeeding helps children attain the necessary nutrients for the first year of their lives (9). Breastfeeding for the initial six months period of life plays an important role in helping the infants to attain optimal growth during their childhood (10).

Breastfeeding aids nutritional benefits and illness protection not only to the infants, but also to the lactating mothers (11). The lactating mothers involved in breastfeeding for longer period are being protected from pregnancy obesity and the risk for cancers in breast and ovaries are observed to be reduced (12). The risk of brittleness in bones leading to osteoporosis was also reported to be lower in mothers who breastfed (3–6 months) their children (13). The risk for immune system mediated diseases and disorders may be decreased by breast milk and breastfed infants, since the breast milk is rich in immunoglobulins that are specific to allergens (14). Thus, we concentrated on reviewing research, especially those including infants with of immune-mediated diseases.

## Materials and methods

### Study design

The database search was carried out by the reviewer on various publication sites such as PubMed, PubMed Central, Nature, Springer, Nature, Web of Science, and Elsevier. The keywords for searching the studies are: breastfeeding, breast milk, human milk, immunity, diabetes, diabetes mellitus, rheumatoid arthritis, diarrhoea, hypersensitivity, allergens, allergic reactions, erythematosus, colitis, hypoglycemia, hyperglycemia, infantile diabetes and protective factor. The duplicate and irrelevant articles were removed, and the data screening was done.

### Inclusion criteria

Only research articles relevant to the current study have been selected. The original research articles, including the *in vivo* studies, were majorly focused, and the studies involving human participants were given higher priority. The recent studies involving human participants with immune-related diseases diagnosis were considered, along with the *in vivo* studies involving the management techniques for immune-related clinical conditions.

Review articles, systematic reviews, and meta-analysis reviews were excluded from the study. The research articles that did not deal with the considered clinical conditions as well as the studies involving *in vitro* analysis were excluded from the study. The articles in which breastfeeding was not associated with the considered immune-related conditions were excluded.

The articles selected on the basis of inclusion and exclusion criteria have been screened manually by the authors for the inter-relationship between breastfeeding and respective clinical conditions. The articles that met the eligible criteria were selected, and data extraction was carried out.

### Data extraction

Preferred Reporting Items for Systematic Reviews and Meta-Analyses (PRISMA) guidelines Liberati et al. (15) were followed for the data extraction procedure. The details of the eligible articles were extracted from the template obtained from the PRISMA website. The details of the included articles contain: (1) year of publication; (2) number of participants; (3) gender of the included participants; (4) age of the participants; (5) race, ethnicity or religion of the participants; (6) immunological disease considered for the analysis; (7) hypothesis framed for the study; (8) methodology used to test the framed hypothesis; and (9) results obtained from the study. A total of 28 articles were considered and presented in the current review.

## Results

**Figure 1:**
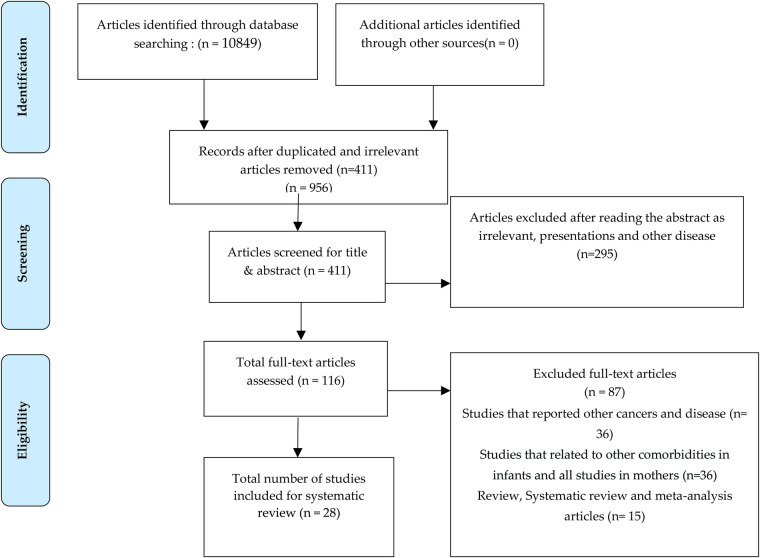
PRISMA flow chart.

Initial screening of articles included around 10,849 articles from the previously mentioned databases and web-sites, from which 411 articles were considered after removing the duplicated and irrelevant articles. Following that, 295 articles were excluded since the presentation of results was found to be irrelevant after reading, and finally 116 articles were fully screened for the current study. After the exclusion of articles based on inclusion and exclusion criteria, 28 research articles were fully analyzed and considered for the review. After screening the abstracts of 116 full-text articles, 87 articles were removed, and 28 articles were found to be in coherence with the current study (Figure 1).

The 28 articles presented in the current review comprise seven studies on “diabetes mellitus with breastfeeding”, two studies about “rheumatoid arthritis and breastfeeding”, five studies about “ role of breastfeeding in celiac disease, twelve studies about allergic/asthma/wheezing conditions and one study on each of the following diseases in association with breastfeeding: neonatal lupus erythematosus and colitis, and. The characteristics of the 28 articles included in the systematic review are summarized in an Appendix in Table A1.

### Breastfeeding and diabetes (type 1 and 2)

Type 1 diabetes is one of the auto-immune diseases that may affect individuals in their early life. It is caused by the autoreactive T cells that ultimately terminate the function of the pancreas's beta cells to produce sufficient insulin. However, its pathological condition may manifest from ten to fourteen years. Nevertheless, its clinical symptoms may occur as early as nine years or as late as 90 years of life (16). Type 2 diabetes is exclusively a metabolic disorder which is meeting the characteristic of type 1 diabetes leads to high blood sugar levels (17). However, the pathogenesis of type 2 diabetes is also documented as an autoimmune disease based on the presence of autoantibodies against beta cells of the pancreas in the blood of people with type 2 diabetes (18, 19).

We have identified six research articles (20) involving the relationship between breastfeeding and diabetes conditions. Three of the studies were reported with type 1 diabetes and rest three studies with type 2 diabetes.

Type 1 diabetes: Two studies were undertaken in Germany, with sample sizes of 990 Rosenbauer et al. (20) and 324 Schaefer-Graf et al. (21). The research that was carried out by Lund-Blix et al. (22) consisted of two population-based cohorts of children who were tracked from the time of their birth (1996–2009) until the year 2014 (in Denmark) or 2015 (Norway), provides evidence in support of the claim that breastfeeding lowers the chance of developing type 1 diabetes.

Type 2 diabetes: Children and adults who received their nutrition from their mothers' breasts rather than from bottles and who were breastfed for longer periods of time throughout infancy had lower rates of type 2 diabetes and lower insulin resistance than those who received their nutrition from bottles (23, 24). It has been hypothesised that variations in the nutritious content of the milk, patterns of baby weight growth, or acquired eating behaviour between infants who are breastfed and babies who are bottle-fed are related to an increased risk of developing diabetes in later life (23). There have only been a few studies that look at diabetes risk in connection to the age at which supplemental meals are introduced to infants (25–27). Longer breast-feeding duration was related with lower fasting insulin concentrations and insulin resistance at 5 years, but not at 9.5 years, according to a research done by S. R. Veena et al. (25). There was no significant relationship found between the age at which a person began eating complementary foods and their glucose or insulin levels. The increased breastfeeding period has been found to be positively associated with the prevention and low risk of type 2 diabetes in lactating mothers Stuebe et al. (26) and children's (27) in the United States population. Similarly, the breastfeeding has also been observed to decrease the risk for type 1 diabetes in females who fed for longer period (22). The susceptibility and possibility of acquiring type 2 diabetes is found to be directly proportional to the period of lactation and breastfeeding in females (28). The presence of a diabetic condition in a breastfeeding female has no effect on the health status of the infants, such as obesity nature or diabetic occurrence (29, 30). Breastfeeding has been reported to be one of the environmental factors that is responsible for children being overweight inversely (30). Reduced breastfeeding has been associated with increased child obesity and type 1 diabetes incidence (31). The risk and occurrence of type 2 diabetes in women may be reduced by suggesting breastfeeding (32). Therefore, breastfeeding plays an important role in protecting infants as well as mothers from the risk of type 1 and type 2 diabetes.

### Rheumatoid arthritis and breastfeeding

Rheumatoid arthritis is a chronic and systemic inflammatory illness that causes irreparable damage to cartilage and bones (33). This damage is caused by inflammation in the synovium of the joints revealed that insulin resistance, a significant contributor to the development of diabetes mellitus, is quite common in people with rheumatoid arthritis (33). In genetically sensitive hosts, environmental stressors may trigger Juvenile idiopathic arthritis (JIA). Shenoi et al. (34) found no link between early infection, prenatal factors, or stressful events. Unfortunately, it has been found that the number of studies that link rheumatoid arthritis and breast milk is significantly low, and the studies that report an association between the two have failed to identify the proper underlying aetiology of rheumatoid arthritis in association with breast milk or breastfeeding in infants (35).

Two studies that show a lower risk of rheumatoid arthritis in children who are breastfed have been taken into consideration in the present systematic review (36, 40). Alotiby, A et al. (36) conducted research that shown the relevance of breast milk to neonates in decreasing the risk of Rheumatoid Arthritis (RA) when compared to formula milk consumption. They investigated the differences in the beginning of the disorder in children who were nursed, children who were not breastfed, and children who were given both breast milk and formula (mixed-fed children). Breastfed children (28.3%), formula-fed children (21.7%), and mixed-fed children (50.0%) were the most common. This difference in feeding method was statistically significant. Formula feeding markedly increased the incidence of RAin children. Hence, exclusive breastfeeding may reduce the risk of RA (36).

The immunological memory of the mother is passed on to her child *via* breast milk, and breast milk includes a range of immune-modulating chemicals, including immune cells and their products such as cytokines (37). Breast milk also allows the mother's immune memory to be passed on to her child. Immunological imprinting and programming of the newborn may be accomplished *via* breastfeeding (37). Therefore, breastfeeding makes a contribution to the development of the immune system of the newborn (38, 39). According to the findings of Kindgren, E. et al. (40), an in-creased risk of juvenile idiopathic arthritis was related with a shorter overall period of breastfeeding as well as a shorter duration of exclusive breastfeeding.

There was an association found between the early introduction of formula (before the age of 4 months) and an elevated incidence of JIA. When potentially confounding factors were taken into account in the model, none of the correlations lacked their statistical significance (40). Breastfeeding may provide some protection against the development of juvenile idiopathic arthritis, according to one finding (41). According to the findings of another re-search, infants who subsequently developed oligoarticular JIA tended to have shorter nursing durations (42). It is recommended that mothers be encouraged to nurse their newborns exclusively for the first four months Kindgren et al. (40), if at all feasible, and then to maintain partial nursing for a prolonged period of time after the introduction of foreign proteins through food.

### Prevention of infantile diarrhea by breastfeeding

It has been shown that beginning breastfeeding as soon as possible and continuing it exclusively protects new-born babies against death due to diarrhea (43). A self-limiting characteristic of the human body that is usually caused by gastroenteritis is termed “diarrhoea.” It is characterized by having loose stools abnormally frequently in a single day (44). The major causes of diarrhoea include dietary habits causing food poisoning or allergies as well as certain medications. We have identified three studies involving the analysis of breastfed infants and their susceptibility to diarrhoea.

We have summarised a study conducted on Qatari children in the year 2009 by Ehlayel et al. (45). The study was targeted at 1,500 mothers and their infants and children aged 1 to 5 years and the response rate was agreed with 1,278 participants. The breastfeeding of the children varied significantly (*p* < 0.001) from 11.4 ± 6.7 months (longer) to 9.2 ± 4.1 months (shorter). In this study, around 11.4% higher risk and susceptibility were observed in the children who received shorter breastfeeding periods, indicating the protective role of breastfeeding against infantile diarrhoea (45).

The other two studies included 93 mother and infant pairs in the Mexican population in the years 2004 and (46, 47). The mean age of the infants was 6 months. The oligosaccharide content present in breast milk influences the diarrhoea in infants and children. The oligosaccharide contents in breast milk were proved to influence the diarrhea in infants (48). The breast milk contains fucosyl oligosaccharides as its major component, and the fucosyl oligosaccharides have a role in controlling diarrhoea in infants in a positive manner *via* innate immune response (47).

The effects of ceasing breastfeeding in the early period and the influence of termination on diarrhoea in infants are adverse. The early termination of breastfeeding increases the risk of infantile diarrhoea (49). Reduced breast-feeding in infants has been positively influenced by the mortality of children along with diarrhoea and other clinical conditions (50).

### Breastfeeding and neonatal lupus erythematosus

A clinical condition caused in infants due to the presence of autoantibodies in lactating mothers is neonatal lupus erythematosus. It is a rare autoimmune disorder (51). Due to the limited number of studies linking breast-feeding and neonatal lupus erythematosus in infants, we have identified one study involving a male infant of 4–6 months. Intense immunoglobulin levels of IgG and IgA were identified in the breast milk, which induced an erythematosus condition in the infant. The antibodies of the lactating mother were found to induce autoimmunity in the infants, and the lesions in neonatal lupus erythematosus conditions were adverse with an increase in breast-feeding (52). They examined the mother's breast milk from an immunological standpoint for their research. Anti-bodies with significant positive IgG and IgA reactivity against nuclear and Ro targets were found in the mother's breast milk, which was a surprise. After this, the doctor recommended stopping nursing, and three weeks later, the lesions disappeared. Since then, the child has been healthy and has not had any diseases. It is possible to draw the conclusion from this that the illness known as neonatal lupus erythematosus is caused by a passive transfer through the placenta of maternal autoantibodies Vanoni et al. (53), the majority of which are directed against the Ro antigen.

### Breastfeeding against colitis

Colitis is a clinical condition in which the large intestine is inflamed. One study has been identified and presented that represents the role of breastfeeding in colitis disease. No studies involving human participants were eligible for the current systematic review, and hence, an *in vivo* study involving interleukin-10 (IL-10) deficient mice is being considered. The duration of breastfeeding as well as breast milk has an impact on the development and progression of colitis inversely (54). Inflammatory bowel disease, a gastrointestinal inflammatory condition that includes Crohn's disease and colitis, is being reduced in infants who were breastfed for a longer period when com-pared to children who had breastfeeding for a shorter period (55).

### Breast feeding and celiac disease

The impact of childhood infections on the development of celiac disease is debatable. Although frequent infections during the first 18 months of life have been linked to an increased risk of celiac disease later in life (56–58), acute infections at the time of gluten introduction have no effect on disease risk in the general population (59). Coeliac disease is multifactorial, resulting from genetic and environmental factors (59). HLA and non-HLA genes are involved, and gluten is a key environmental factor because the disease remits when gluten is eliminated. The important case-control study by Ivarsson et al. 2002 concludes that breast milk protects under-2-year-olds from coeliac disease (60).Different studies such as case control, follow-up studies, comparative studies showed a significant correlation between breast feeding and coeliac disease (60–63). Breastfeeding (62, 64) and later gluten introduction (61, 63) reduced celiac disease incidence. Different populations had delayed celiac disease onset (65–67). Elena Lionetti (68) reported the administration of gluten in the early of life was linked to a development of illness in the later stage of life.

### Role of breastfeeding in hypersensitivity and allergic conditions

An abnormal or altered immunological reaction that is in response to the untimely response of the immune system is termed hypersensitivity (69). The hypersensitivity or allergic reactions is majorly targeted towards harmless foreign substances resulting in damage of tissues (69). One study regarding hypersensitive and allergic reactions has been included in this current review, which includes the screening of 1,278 lactating Qatari mothers and their infants and children (70). The mean age of the participating mothers was 32.5 years, and the children were 2.5 years old. The period of the study was from around the years 2006 to 2007. More than 59% of infants were exclusively breastfed, 28% were partially breastfed, and the remaining infants were not breastfed. The study report revealed a significant variation (*p* < 0.01) in the occurrence of allergic reactions (70). Allergy and hypersensitive reactions are prevented in infants receiving breast milk, indicating the protective nature of breast milk against hypersensitivity and allergic reactions (71–80).

A respiratory condition called asthma is being triggered by the immune cells as the result of allergic response to certain environmental factors (70). The breastfeeding influences the risk of developing asthma (81). According to Malcolm Sears et al. (82), nursing does not prevent children from atopy or asthma and may potentially increase the risk. Breast milk has been shown to transport food molecules intact from the maternal body to the infants (81). A study has reported hypersensitive allergic reactions towards fish by infants due to increased dietary fish intake by the mother (83). Similar allergic reactions have also been observed in infants in response to egg intake as well as peanut intake by lactating and breastfeeding mothers (84).

## Discussion

This structured and systematic review on breastfeeding as a protective role against the development of auto-immune diseases identified around 20 relevant and appropriate articles related to breastfeeding and autoimmune diseases. Most of the considered investigations were cohort and follow-up studies on lactating mothers and breast-feeding infants. Many research articles were published in accordance with breastfeeding and its protective role against immune-mediated diseases. The objectives of interest were breastfeeding, ingestion of breast milk, and immune-related diseases in infants. It has been reported that the breast milk of diabetic mothers fed to their infants for a longer period as well as in larger volumes induces childhood obesity (85). But, in this review, we have suggested and provided evidence that childhood overweight as well as childhood obesity is prevented by longer breastfeeding than partial breastfeeding. The authors would also like to add the fact that the glucose content in breast milk of diabetic and non-diabetic mothers is similar (86).

Few studies have been reported in favour of breastfeeding as a protective factor against rheumatoid arthritis, which is currently being discussed in this review. Children's exposure to breastfeeding for less than four months increases the risk of rheumatoid arthritis during their childhood stage (87). Middle-aged and elderly women who had been breastfeeding their children for an extended period were less prone to rheumatoid arthritis, and breast-feeding is observed to be positively associated with the prevention of rheumatoid arthritis in infants during their childhood and in mothers at their elderly age (35). The relationship between breastfeeding and rheumatoid arthritis have concluded that the breastfeeding eventually reduces the susceptibility and risk of rheumatoid arthritis irrespective of feeding period (88).

A skin condition called eczema has been reported as one of the allergic conditions prevalent in early childhood as well as in infants and has been associated with the food intake of the lactating mother (89). Asthma and other respiratory infections in the early stages of a child's life have been found to be associated with breastfeeding inversely, i.e., the longer the breastfeeding, the lower the risk of respiratory infections (90, 91). An allergic reaction in the nose resulting in rhinitis has been reported to be reduced in breastfed children Bloch et al. (92). A hypothetical suggestion is to be provided in the case of allergic and hypersensitivity reactions to breastfeeding, since breastfeeding may induce hypersensitive reactions in some cases as well as protection in a few cases, as reported in this review.

A few studies have reported that the diabetic condition, accompanied by a gluten intolerance clinical condition, celiac disease, is being influenced by breastfeeding (93). The risk for developing autoimmune nature in the infants increases when the microbial infections are diagnosed before 9th month of their life (94). Here, the authors present a contrasting review that shows autoimmune conditions are prevented and protected in children who were breastfed for more than 4 months. Breastfeeding favours the immune system of infants to produce necessary immunity (95). Infants fed with breast milk are being observed to attain support for their immune systems that has yet to be matured. The mechanism involves the components of breast milk like anti-inflammatory cytokines, which is an immune-modulating compound (96).

At birth, a newborn infant is immediately exposed to a vast array of microbes from the environment, but primarily from the mother (97). However, breast milk then “feeds” the gastrointestinal tract both directly (maternal milk microbes) and indirectly (through the birth process) (97). In breastfed infants, the microbiome predominantly consists of Bifidobacterium (B breve, B longum, and B bifidum) (98). In contrast, the microbiome of formula-fed infants is more diverse, with the increased relative abundance of Bacteroidetes and Firmicutes, and with increased Clostridium difficile (98). In contrast, the microbiome of formula-fed infants is more diverse, with an increased relative abundance of Bacteroidetes and Firmicutes (99). There is a complex relationship between breast milk and the infant's microbiome (98). This relationship involves the transfer of immunoglobulins, bacteria, viruses, and bacteriophages (viruses that parasitize a bacterium by infecting it and reproducing inside it) from the mother to the infant through the mother's only milk. The microbiome begins to resemble that of an adult by the third year of life, and the sequential acquisition of gut microbes early in life has a long-lasting effect on gut health. This occurs as the micro-biome gradually transforms to match that of an adult. A disruption in the establishment of this microbiome has been linked to increased risks of obesity, diabetes, and mental health disorders, as well as immune-mediated and inflammatory conditions such as inflammatory bowel disease and atrophy. Breastfeeding an infant is related to a lower risk of diarrhoeal illness than formula-feeding an infant. The variations in the microbiota of breastfed and formula-fed infants continue to exist beyond six months (100, 101). The current review also reported a similar suggestion that breastfeeding favours the maturation of the immune system in infants.

## Conclusions

Based on all the literature surveyed for the current systematic review, we conclude that breastfeeding infants anonymously helps them to build a mature, strong, and healthy immune system against immunological conditions. The breastfeeding helps the infants to protect against certain acquired immunological conditions.

## Data Availability

The original contributions presented in the study are included in the article/Supplementary Material, further inquiries can be directed to the corresponding author/s.

## Appendix A

**Table A1:** Characteristics of the included studies.

Sl.no	Name of the Authors	Gender	Infant /Maternal focused study	Number of Study Participants	Age Group	Ethnicity / Race / Study Region	Type of Disease	Hypothesis Framed	Methodology Used	The outcome of the Study
1	Alotiby et al. (36)	Male - 29; Female – 31	Infant	60	1 to 16 years	Saudi Arabia	Rheumatoid Arthritis and Inflammatory Bowel Disease	To determine the relationship of breastfeeding with Rheumatoid Arthritis and Inflammatory Bowel Disease	A cross-sectional study	Breastfeeding reduces the risk of Rheumatoid Arthritis in infants
2	Kindgren et al. (40)	Not disclosed	Infant	10,565	0 to 18 years	Swedish	Rheumatoid Arthritis	To study the influence of early breastfeeding on later development of Rheumatoid Arthritis	A case control study in a prospective birth cohort	Longer breastfeeding reduces the risk for Rheumatoid Arthritis in the later age of childrens
3	Kemppainen et al. (61)	Not disclosed	Infant	6,327	1 to 4 years	United States and Europe	Celiac Disease	To determine the relationship of breastfeeding with celiac disease	A follow-up study	Breastfeeding reduces the risk for celiac disease
4	Lund-Blix et al. (22)	Not disclosed	Infant	504	6 to 18 months	Norwagean and Danish	Type 1 Diabetes	To find the relation between type 1 diabetes and breastfeeding	Two population based cohort and follow-up study	Breastfeeding reduces incidence of type 1 diabetes
5	Shenoi et al. (34)	Male - 126; Female - 237	Infant	363	8 to 15 years	Not Disclosed	Juvenile Idiopathic Arthritis	To investigate the association of breastfeeding and development of Juvenile Idiopathic Arthritis	Case-control study	no association was observed
6	Palma et al. (62)	not disclosed	Infant	164	1 to 4 months	Spain	Celiac Disease	To find whether breastfeeding protect children from developing celiac disease	PROFICEL study	Breastfeeding has a protective role against celiac disease
7	Veena et al. (25)	Not disclosed	Infant	518	3 months to 18 months	India	Diabetes mellitus	To examine whether breast-feeding is associated with lower glucose concentrations and insulin resistance	Follow-up study	Longer duration of breastfeeding protects against glucose intolerance and insulin resistance in children
8	Mayer Davis et al. (27)	Female	Infant	247	10 to 21 years	African Americans	Type 2 Diabetes	To evaluate the association of breast-feeding with type 2 diabetes incidence	Case control study	Breastfeeding is protective against development of type 2 diabetes in youth
9	Kristiana Gray et al. (52)	Male	Infant	1	4 months	United Kingdom	Neonatal lupus erythematosus	To predict the breast milk and lupus interaction	Single case study	Breast milk contained immunoglobin increasing the lupus lesions
10	Bener et al. (70)	Not disclosed	Infant	1,278	0 to 5 years	Qatari children	Allergic diseases	To assess the relationship between breastfeeding and the development of childhood asthma and allergic diseases	A cross sectional study	Breastfeeding prevents asthma and hypersensitivity reactions
11	Rosenbauer et al. (20)	Not disclosed	Infant	990	Less than 5 years	Germany	Type 1 Diabetes	To evaluate the association between type 1 diabetes risk and breastfeeding in preschool children	Case-control study	Breastfeeding prevents the development of type 1 diabetes
12	Schaefer-Graf et al. (21)	Male - 175; Female - 149	Infant	324	Children (Not disclosed)	German	Type 1 Diabetes	To determine the association of breast-feeding and early childhood overweight in children from mothers with gestational diabetes mellitus	Follow-up demographic study	Breastfeeding >3 months: does not cause infant overweight; Brestfeeding <3 months: associated with infant overweight
13	Young et al. (32)	Not disclosed	Infant	138	<18 years	Native Canadian	Type 2 Diabetes	To determine whether breastfeeding reduces the risk of type 2 diabetes among Native Canadian children	Case-control study	Breastfeeding reduces the risk of type 2 diabetes in Infant
14	Ivarsson et al. (60)	Not disclosed	Infant	1,881	0 to 2 years	Swedish	Celiac Disease	To compare the effects of breast feeding on risk of celiac disease	A population-based incident case-referent study	Breastfeeding reduces the risk for celiac disease
15	Sollid L.M. et al. (63)	Not disclosed	Infant/Child	1,881	1 to 15 years	Swedish	Celiac Disease	To find whether breastfeeding protect children from developing celiac disease	population based case control study	breastfeeding reduces the risk of celiac disease in early childhood and also during the subsequent childhood period.
16	Elena Lionetti (68)	Not disclosed	Infant/Child	832	6 months to 10 years	Italy	Celiac Disease	To determine the facor of gluten in the diet of infants for the development of celiac diasease with their age	Comparative study	Gluten diet did not affect for the development of the diseases in infant stage but made a significant difference by the development of the diseases in later of their life.
17	Malcolm Sears et al. (82)	Not disclosed	Infant	1,037	3 years	Newzealand	Asthma and Atopy	To assess long-term outcomes of asthma and atopy related to breastfeeding	Birth cohort study	Breastfeeding does not protect children against atopy and asthma and may even increase the risk
18	U. Peters et al. (64)	Not disclosed	Infant	280	1 to 10 years	Germany	Celiac Disease	To investigate the association between the duration of breast-feeding and celiac disease	Case-control study	Breastfeeding has a significant protective effect on the incidence of celiac disease
19	Yamakawa, M et al. (71)	Male-18 186	Infant	35,215	6 to 42 months	Japan	Asthma	The associations between breast-feeding and hospitalization for asthma in early childhood	longitudinal survey	Beneficial effects of breast feeding on hospitalization for asthma in early childhood.
		Female-17 029								
20	M.B. Azad et al. (72)	Not disclosed	Infant	2,773	3 to 12 months	Canada	Wheezing	association of breastfeeding and wheezing in the first year of life	Longitudinal study	Breastfeeding appears to confer protection against wheezing in a dose-dependent manner among infants born to mothers with asthma
21	van Meel, E.R et al. (73)	Not disclosed	Infant	4,464	Birth until 1 year of age	Netherlands	Asthma	To examine the associations of breastfeeding, with lung function and asthma	population-based prospective cohort study	Shorter duration and non exclusivity of breastfeeding were associated with a lower forced expiratory volume (FEV1) and forced vital capacity (FVC) but not asthma
22	Huang, C et al. (74)	Male-18 6,536	Infant	13,289	4–6 year	Shanghai (China)	Asthma, Allergies, and Airway Diseases	Associations of breastfeeding durations with prevalences of asthma, wheeze, hay fever, rhinitis, pneumonia, and eczema among preschool children	cross-sectional study	Findings support China‟s national recommendation that mothers provide exclusive BF for the first four months, and continue partial BF for more than six months.
		Female-6,753								
23	Peters, R.L et al. (75)	Not disclosed	Infant	3,663	1 year (followed for 6 years)	Australia	Asthma	Relationship between breastfeeding ever and duration on the 24 development of asthma and allergic asthma phenotypes	A population-based, longitudinal study	Longer duration of breastfeeding was associated with a reduced odds of asthma among children without eczema in the first year of life
24	Al-Makoshi, A et al. (76)	Male-327	Infant	622	1–3-year	Riyadh, Central Saudi Arabia	wheezing/asthma and atopic disease	Investigated the prevalence of breastfeeding and its association with wheezing/asthma and atopic disease	A cross-sectional study	Full breastfeeding is associated with reduced childhood wheezing and possibly asthma
		Female-295								
25	Qu, F et al. (77)	Not clear	Infant	5,479	3–6 years	Beijing, China	asthma and allergy	prevalence and risk factors for asthma, allergy and related symptoms; and breastfeeding patterns and durations	A cross-sectional study	“More” breastfeeding appears to be protective against asthma and related symptoms
26	Hu, Y et al. (78)	Male- 5464	Infant	10,464	6–11 years	Shanghai, China	Asthma and allergic	Examined whether breastfeeding modifed the efects of neonatal and familial risk factors on childhood asthma and allergic diseases	A population-based study	Longer breastfeeding duration was inversely associated with childhood Asthma and allergic diseases, and also reduced the OR of neonatal and familial risk factors on these diseases
		Female- 5,000								
27	Ehlayel, M.S. and Bener, A. (79)	Male- 632	Infant	1,278	0 –5 years	Qatar	Allergic diseases	Assess the effect of Exclusive breast-feeding on the development of allergic diseases and eczema	A cross sectional study	Prolonged breast-feeding reduces the risk of developing allergic diseases and eczema even in the presence of maternal allergy
		Female- 646								
28	Obihara, C.C et al. (80)	Male-438	Infant	861	6–14 Years	Cape Town, South Africa	Allergic diseases	Association between allergic disease in children and prolonged breastfeeding	A cross sectional study	Protective effect of prolonged breastfeeding on the development of allergic disease, particularly hay fever, in children born to nonallergic parents
		Female- 423								
